# ApoC3 is expressed in oocytes and increased expression is associated with PCOS progression

**DOI:** 10.1186/s13048-023-01263-6

**Published:** 2023-09-09

**Authors:** Jiahe Zhou, Hui Mo, Qian Feng, Li Li, Jiahui La

**Affiliations:** 1 Guangdong Women and Children Hospital, Guangzhou, 511442 China; 2 Guangzhou Medical University, Guangzhou, 511436 China; 3 Faculty of Chinese Medicines, Macau University of Science and Technology, Macao, 000853 China; 4 International Institute for Translational Chinese Medicine, Guangzhou University of Chinese Medicine, Guangzhou, 510006 China

**Keywords:** Polycystic ovary syndrome, Apolipoprotein C3, Ovary, Oocyte

## Abstract

**Background:**

Polycystic ovary syndrome (PCOS) is a lifelong metabolic disorder and the most common cause of anovulatory infertility affecting women in reproductive age. Our recent study reported that apolipoprotein C3 (ApoC3) could be a potential diagnostic serum marker for metabolism disturbance in PCOS patients, but whether it is present in the ovaries and what role it plays has not yet been described.

**Objective:**

Aimed to investigate ApoC3 expression in ovary of PCOS, and to discuss its potential role in PCOS progression.

**Methods:**

ApoC3 expression in ovarian tissue samples from 12 PCOS patients along with 12 healthy controls were measured via immunohistochemistry (IHC). Also, the level of ApoC3 in follicular fluid from 14 patients diagnosed with PCOS and 13 control subjects were detected by ELISA. The expression and location of ApoC3 in ovaries of PCOS mice were tested weekly for three consecutive weeks during PCOS formation using real time PCR, Western Blot, IHC and immunofluorescence. The relation of ApoC3 and sex hormones was analyzed in mouse plasma. Additionally, the dynamic changes of ApoC3 level in ovaries of healthy mice during postnatal development was also investigated.

**Results:**

ApoC3 levels in ovarian tissue and follicular fluid were significantly higher in PCOS patients than in controls (33.87 ± 4.11 vs. 27.71 ± 3.65, *P < 0.01*; 0.87 ± 0.09 vs. 0.51 ± 0.32 ng/mL, *P < 0.05*), respectively. In ovary, ApoC3 was found to be located in the cytoplasm of oocyte, and its expression gradually increased with PCOS progression (*P < 0.05*). Furthermore, correlation analysis showed that plasma ApoC3 level was closely associated with luteinizing hormone (*r = 0.709, P = 0.001*), testosterone (*r = 0.627, P = 0.005*) and anti-mullerian hormone (*r = 0.680, P = 0.002*) in PCOS mice. In addition, ApoC3 level in oocyte was physiologically increased and peaked on postnatal age 21 (P21), then decreased following P21 in healthy mice.

**Conclusions:**

We identified ApoC3 expression in oocyte. It may be involved in PCOS progression and possibly participate in the regulation of oocyte development.

**Supplementary Information:**

The online version contains supplementary material available at 10.1186/s13048-023-01263-6.

## Introduction

PCOS is one of the main causes for anovulatory infertility, which affects 6–20% of women in reproductive age worldwide. In women with PCOS, there are about 75% suffer from anovulation infertility and 50% experience with recurrent pregnancy loss [1–3]. PCOS is a multi-factorial and heterogeneous syndrome characterized by excessive androgens, ovulatory dysfunction, and polycystic ovarian morphology, and is often accompanied with metabolic disorders, such as insulin resistance (IR), abnormal lipid metabolism, metabolic syndrome, type 2 diabetes and cardiovascular disease. Its impact on women’s physical and mental health has made it a severe public health challenge [4–8].

Recently, we reported that serum samples from women with PCOS contained an abnormally high level of ApoC3 [9]. ApoC3 is a smaller secreted glycoprotein of 79 amino acid residues predominantly produced in the liver, and plays an important role in lipid metabolism [10]. ApoC3 inhibits lipoprotein lipases and prevents ApoB and ApoE apolipoproteins from interacting with their hepatic receptors, thereby increasing the bioavailability of circulating TG-rich lipoproteins via reducing lipolysis and therefore reduces hepatic uptake. Increasing level of circulating ApoC3 has widely been proposed as a biomarker for hypertriglyceridemia and metabolic imbalance in type 1 diabetes, chronic kidney disease and acute lymphoblastic leukemia [11–13]. It has been established that PCOS patients may have an increased risk of hyperlipemia and metabolic diseases, but rare study has been conducted to investigate the relationship between ApoC3 and PCOS [14].

Ovaries are the female reproductive organs and produce ovum and sex hormones (androgens, estrogen, and progesterone) upon stimulation by gonadotropins [15]. Pathological ovarian follicular development is the primary characteristic mediated by various subtle mechanisms as well as metabolic and intraovarian interactions in the anovulation of PCOS [16]. In PCOS, the accumulation of prematurely arrested small antral follicles within the ovarian cortex and subsequent failure of dominant follicle development result in PCOM [17]. The aforementioned follicular arrest in PCOS is clinically manifested in menstrual irregularity and anovulation [18]. There is a close relationship between lipid metabolism and ovarian function, and research in this area has received increasing attention in recent years. Previous study has shown that metabolism of lipoproteins during steroidogenesis in oogenesis and follicular development for ovulation is highly complex and involves several factors depending on the follicular stage [19].

In the present research, ovary samples and follicular fluid of PCOS patients were collected and determined to check the expression of ApoC3, and compared to those in control women. Furthermore, A PCOS mouse model was developed by intraperitoneal injections of dehydroepiandrosterone (DHEA) for a consecutive three weeks and investigated weekly to determine the role of ApoC3 and its contribution to the ovarian dysfunction of PCOS. Moreover, the fluctuation of ApoC3 levels in mouse ovary during pubertal development was also observed using mouse in postnatal age. The purpose of this study was to assess the relationship between ovarian ApoC3 and PCOS progression, and to provide a useful clue for elucidating the molecular mechanisms of etiology in ovulation disorder of PCOS.

## Results

### ApoC3 level was increased in the ovarian tissues and follicular fluid from women with PCOS

**Fig. 1:**
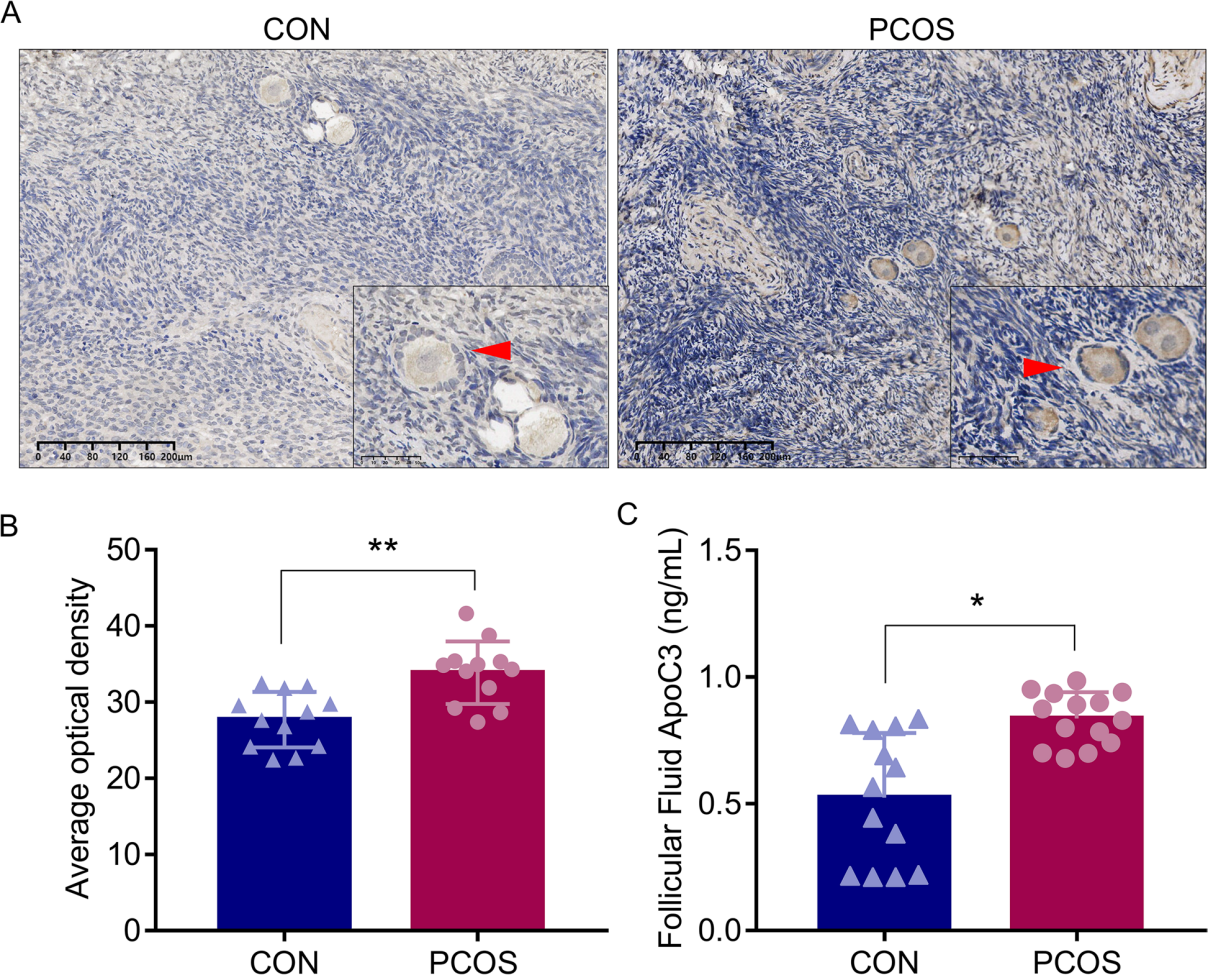
ApoC3 expression increased in ovarian tissue and follicular fluid of PCOS patients. **A** Immunohistochemical pictures of ovary in PCOS patients (*n* = 12) and non-PCOS patients (*n* = 12), scale bars: 200 μm, 50 μm. Area positive for ApoC3 was stained in brown; oocytes were indicated by red arrowheads. **B** Average optical density of ApoC3. **C** ApoC3 levels in follicular fluid from PCOS patients (*n* = 14) and controls (*n* = 13). Data were shown as mean ± SD. *P < 0.05, **P < 0.01 versus control group

A total of 12 paired ovary samples from PCOS patients based on the Rotterdam criteria and controls were selected by matching age and BMI (Table 1). As shown in Fig. 1A, the average optical density (AOD) of ApoC3 was significantly elevated in PCOS ovarian tissues versus control group (33.87 ± 4.11 vs 27.71 ± 3.65, *P < 0.01*) (Fig. 1B). We then further analyzed the expression of ApoC3 in follicular fluid, and found a significantly increased level of ApoC3 in follicular fluid of PCOS group than the control group (0.87 ± 0.09 vs 0.51 ± 0.32 ng/mL, *P < 0.05*) (Fig. 1C*).*

**Table 1:** Clinical data from ovarian specimens and follicular fluid in control and PCOS patients (X ±S)

Parameter	Ovarian tissue		Follicular fluid	
	Control (*n* = 12)	PCOS (*n* = 12)	Control (*n* = 13)	PCOS (*n* = 14)
Age (years)	28.83 ± 4.91	27.83 ± 3.30	28.33 ± 3.82	29.33 ± 3.82
BMI (kg/m^2^)	25.17 ± 3.28	27.13 ± 3.96	21.71 ± 4.66	21.80 ± 4.23
LH (IU/L)	6.08 ± 2.46	10.92 ± 4.64*	5.60 ± 2.02	7.03 ± 2.35
FSH (IU/L)	5.71 ± 1.29	5.96 ± 1.23	6.87 ± 1.74	5.52 ± 1.36
T (nmol/L)	1.22 ± 0.19	2.08 ± 0.80**	1.26 ± 0.06	1.65 ± 0.45*

Data were presented as mean ± SD. Mean ± SD are shown. The Student’s t test was used for normally distributed data

*BMI* Body mass index; *LH* Lutein stimulating hormone; *FSH* Follicle stimulating hormone; *T* Testosterone

*P < 0.05, **P < 0.01 versus control group

### PCOS-like phenotypes in DHEA-treated mice

**Fig. 2:**
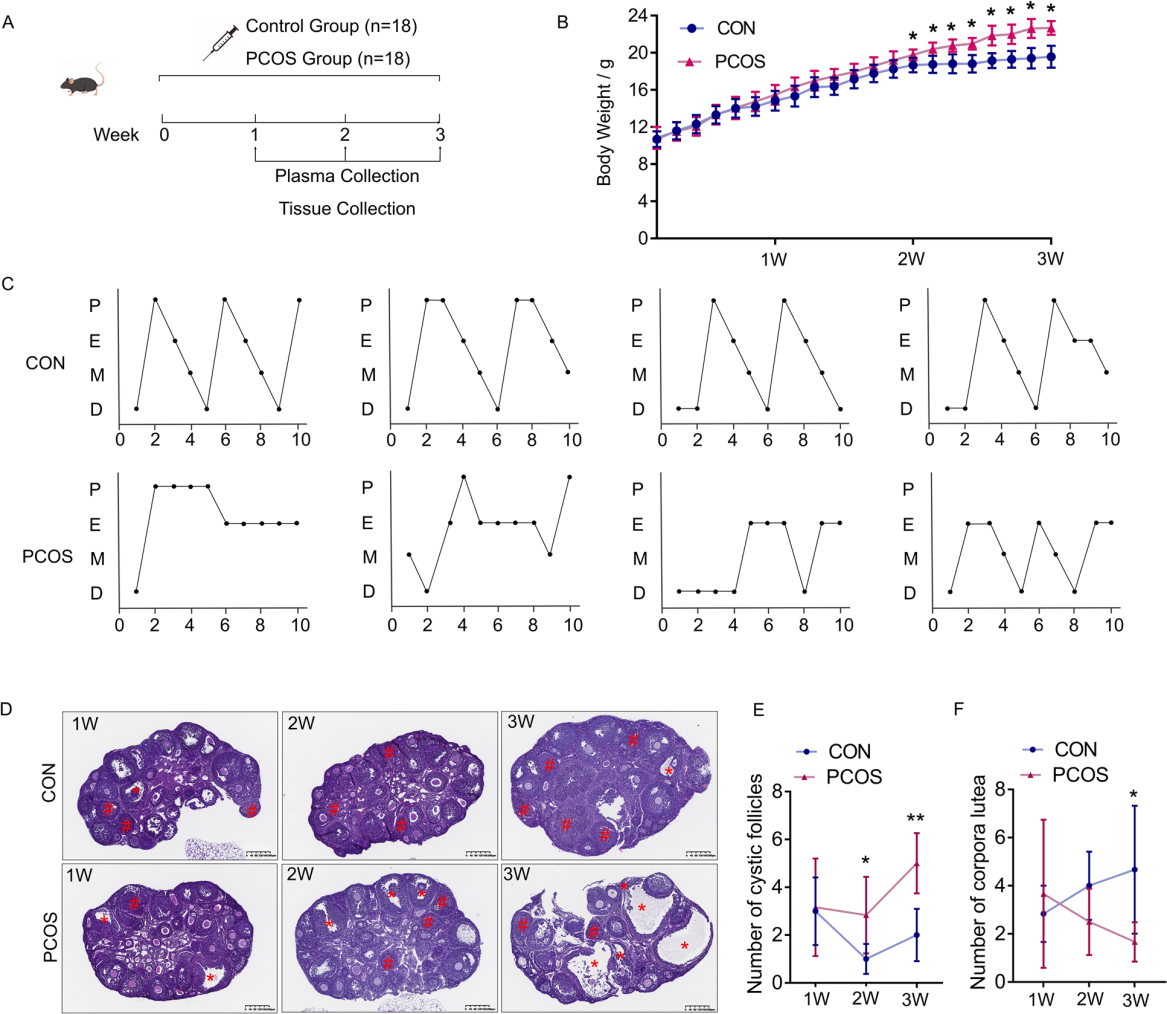
Impairment of ovulatory function in mouse models for PCOS. Mice were injected with DHEA decoction (6 mg/100 g.d) and sesame oil for consecutive 3 weeks and examination weekly. **A** Schematic illustration of the experimental design of PCOS mouse models. **B** Body weight. **C** Representative oestrous cycle of one mouse from each group. P, proestrus; E, estrus; M, metestrus; D, diestrus. **D** Representative HE staining of ovarian tissue from one mouse from each group. The cystic follicle is indicated by red asterisks; corpora lutea was indicated by red pound signs. Scale bar: 200 μm. **E** Quantitative analysis of cystic follicles (*n* = 6). **F** Quantitative analysis of corpora lutea (*n* = 6). Line graphs show the mean ± SD. ***P < 0.001, **P < 0.01, *P < 0.05 versus control group

**Fig. 3:**
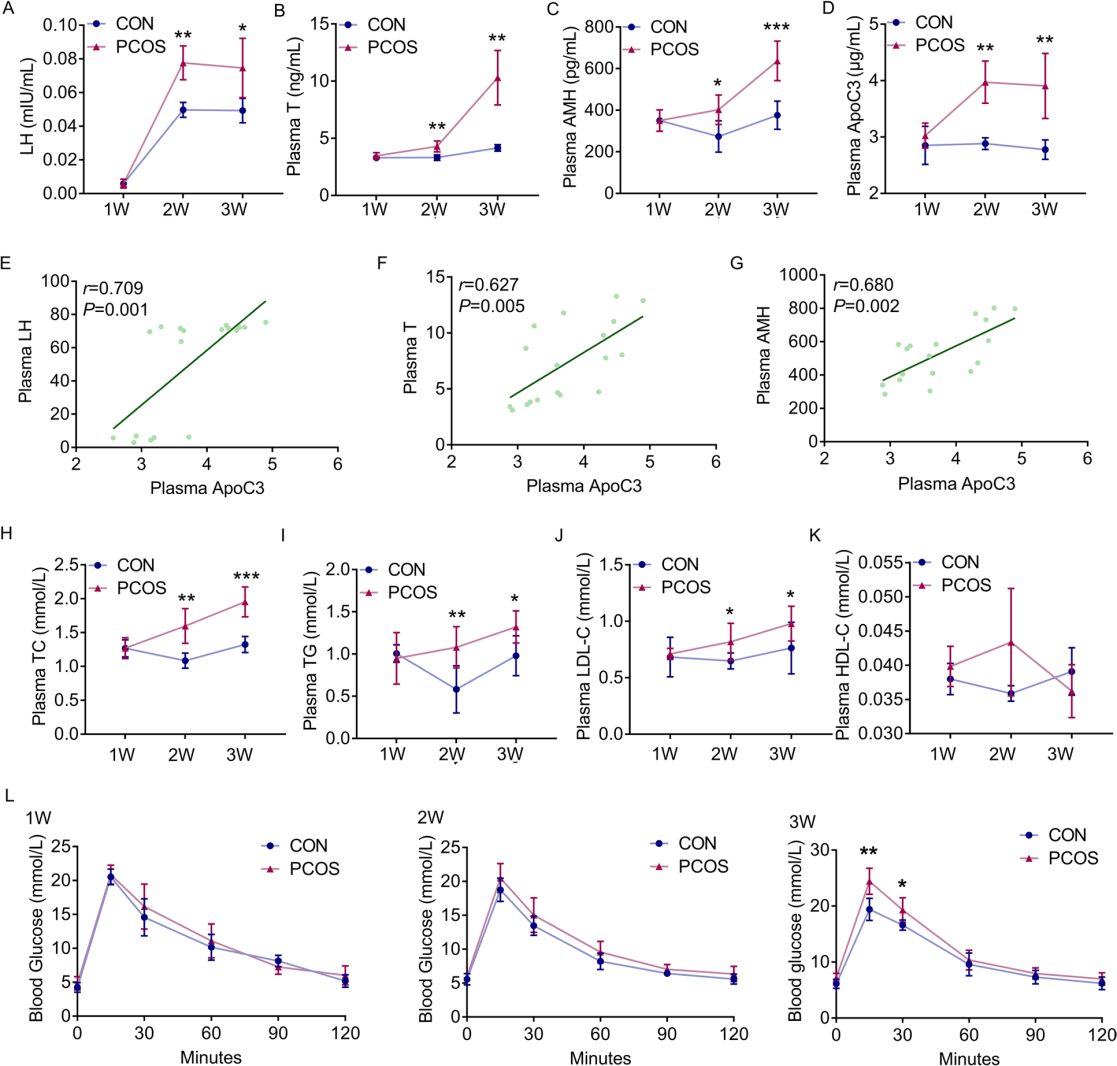
ApoC3 was increased and associated with sex hormone in PCOS mouse model. **A** Plasma level of lutein stimulating hormone (LH); (**B**) Plasma level of testosterone (T); (**C**) Plasma level of anti-mullerian hormone (AMH). **D** Plasma level of ApoC3. **E** Correlation analysis between ApoC3 and T in PCOS group (*n* = 18), *r* = 0.709 *P* = 0.001. **F** Correlation analysis between ApoC3 and AMH in PCOS group (*n* = 18), *r* = 0.627 *P* = 0.005 (*n* = 18). **G** Correlation analysis between ApoC3 and LH in PCOS group (*n* = 18), *r* = 0.680 *P* = 0.002. The lines indicate the fitted regression curves. **H** Total cholesterol (TC). **I** Total triglycerides (TG). **J** Low density lipoprotein cholesterin (LDL-C). **K** High density lipoprotein cholesterin (HDL-C). **L** Blood glucose level by GTT (*n* = 6 each group). Line graphs show the mean ± SD. ***P < 0.001, **P < 0.01, *P < 0.05 versus control group

A PCOS-like mouse model was established via DHEA treatment as previously described [20, 21]. It was found that the body weights of PCOS mice were significantly increased at Week 2 compared to the control group (19.80±0.79 vs 18.62±0.67 g, *P*<0.05, Fig. 2B). The mouse model for PCOS showed disrupted estrus cycles, while the cycles of the control group remained regular (Fig. 2C). Furthermore, histological analysis of PCOS mouse ovaries found more cystic follicles and fewer corpora lutea when compared to control ones from Week 2 (2.83±1.60 vs 1.00±0.63, *P*<0.05; 2.17±1.17 vs 3.67±1.63, *P*>0.05, Fig. 2D-F). The plasma levels of luteinizing hormone, testosterone and anti-mullerian hormone, which were known to be associated with PCOS development [22], were obviously elevated in PCOS mice at Week 2 (0.078±0.010 vs 0.050±0.004 mIU/mL, *P*<0.05; 4.30±0.48 vs 3.32±0.24 ng/mL, *P*<0.01; 402.55±70.74 vs 273.62±75.35 pg/mL, *P*<0.05, Fig. 3A-C). Aside from a significant rise in sex hormones, abnormal glucose and lipid metabolism were also observed in mouse models for PCOS (*P*<0.05, Fig. 3H-L).

### Correlation between ApoC3 and sex hormones in PCOS mice

Noteworthy, we uncovered a significant and persistent elevation in circulating levels of ApoC3 in plasma of PCOS mice at Week 2 compared to the control group (3.97±0.37 vs 2.88±0.10 μg/mL, *P*<0.01, Fig. 3D). To verify the association between ApoC3 and the endocrine profiles, linear regression analyses were performed. Correlation analysis showed a positive relationship between ApoC3 and luteinizing hormone (*r*=0.709, *P*<0.01), testosterone (*r*=0.627, *P*<0.01) and anti-mullerian hormone (*r*=0.680, *P*<0.01) in mouse models for PCOS (Fig. 3E-G, Table S1). In contrast, no correlation was found between ApoC3 and hormone profiles in the control group (*P*>0.05, Table S2).

### ApoC3 gradually increased with PCOS progression in mouse model and located in oocyte

**Fig. 4:**
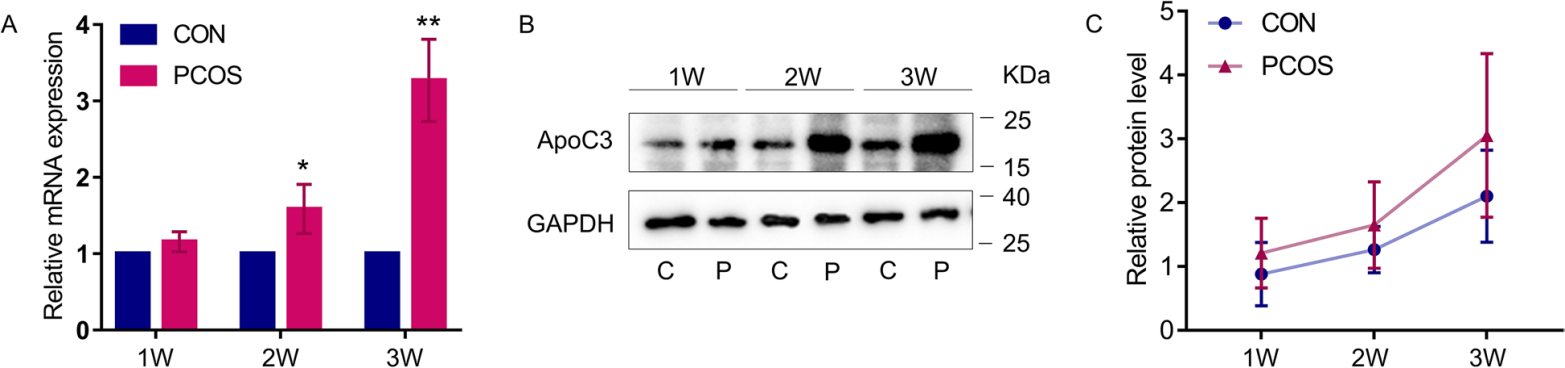
Overexpression of ApoC3 in ovaries for PCOS mice was detected by Western blot and RT-PCR. ApoC3 expression demonstrated by real-time qPCR and western blot at mRNA (**B**) and protein (**C**) levels (*n* = 3). (C) The relative protein expression of ApoC3. Data are expressed as mean ± SD, *P < 0.05, **P < 0.01 versus control group

**Fig. 5:**
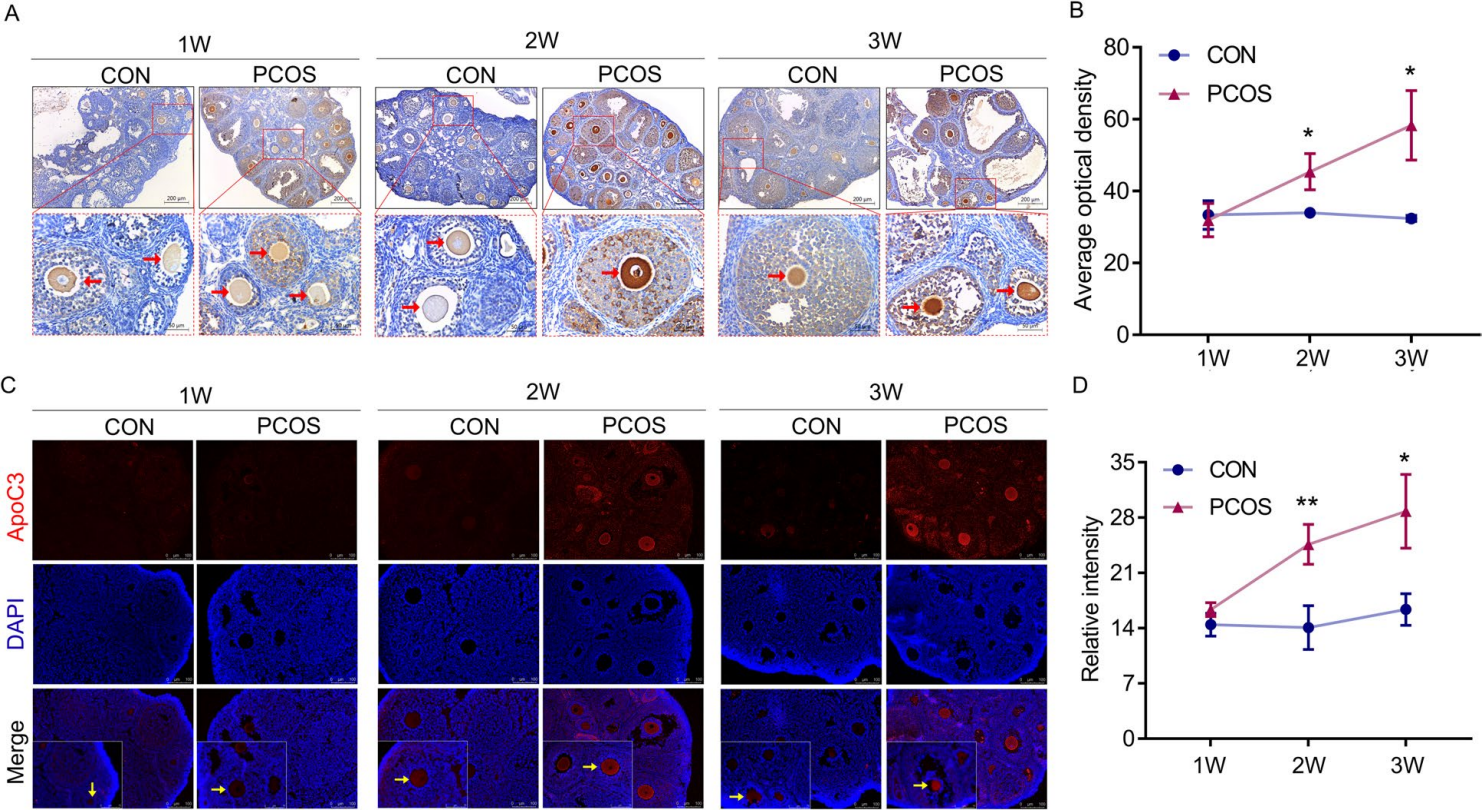
Expression of ApoC3 was increased significantly in oocyte of PCOS mice with the time of disease progress. **A** The Immunohistochemical staining (*n* = 3). Positive for ApoC3 was stained in brown; oocytes were indicated by red arrows. Scale bar: 200 μm, 50 μm. **C** Immunofluorescence staining (*n* = 3). Red, ApoC3. Blue, DAPI indicates the nuclear localization signal. Scale bar: 100 μm, 75 μm. Oocytes were indicated by red arrows. Data are expressed as mean ± SD, *P < 0.05, **P < 0.01 versus control group

qPCR and western blot were used to examine ApoC3 expression in the mouse ovarian tissues, and the results indicated that ApoC3 mRNA and protein levels in the ovaries were increased in the PCOS mice from Week 2, compared with the control mice (Fig. 4), and the expression of ApoC3 was increased with progression of PCOS. Immunohistochemical and Immunofluorescence stain showed that ApoC3 was mainly expressed in the oocytes, with higher levels in PCOS mice compared with the controls (*P*<0.05, Fig. 5), which demonstrated that the source of ApoC3 may be from oocyte.

**Fig. 6:**
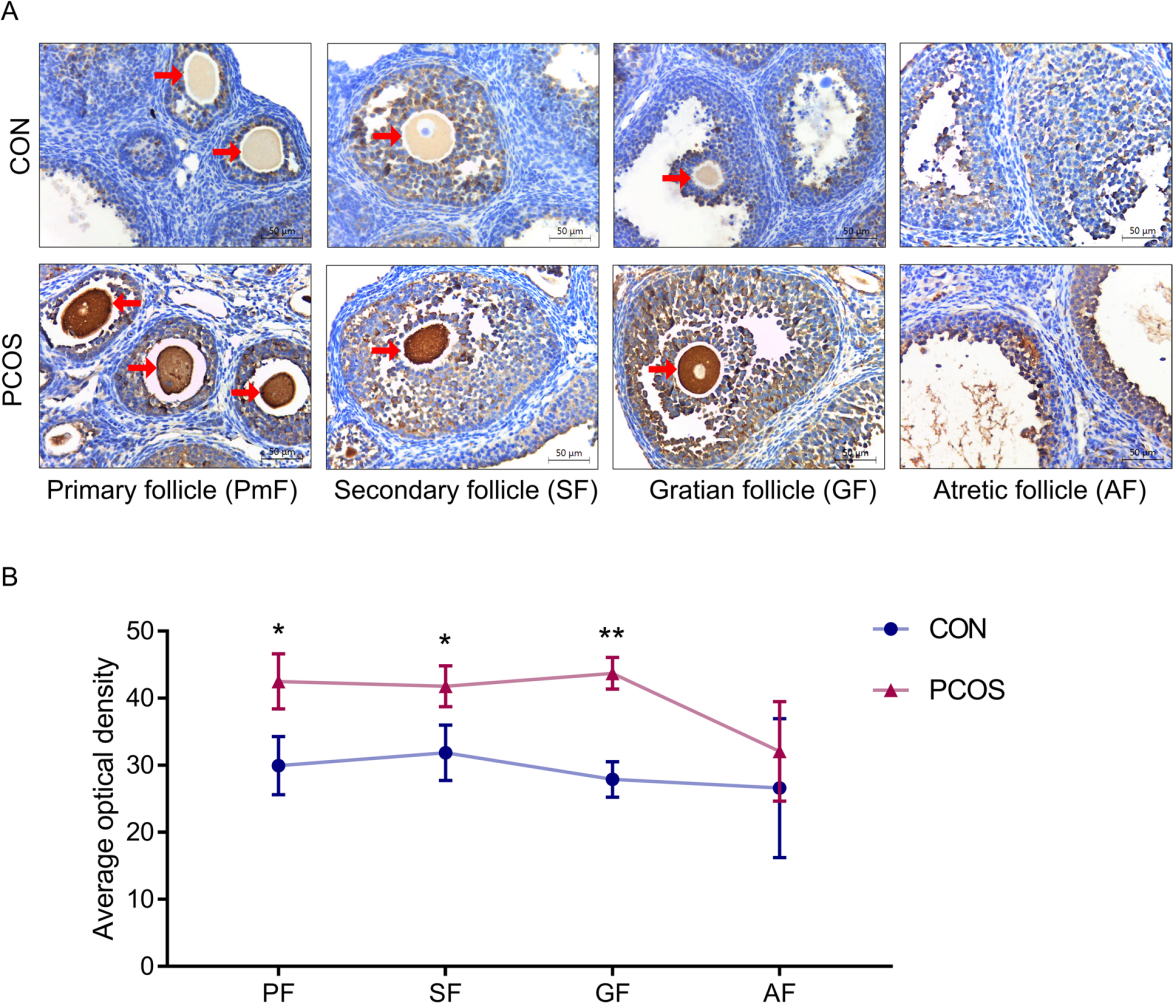
ApoC3 was highly expressed in oocytes in different follicular stages of PCOS mice (**A**) Immunohistochemical staining of ApoC3 in four developmental stages of follicles. The area positive for ApoC3 is stained in brown; oocytes were indicated by red arrows. Scale bar: 50 μm. **B** Average optical density of ApoC3. Primary follicle (PF), secondary follicle (SF), Gratian follicle (GF), Atretic follicle (AF). Data are expressed as mean ± SD, *P < 0.05, **P < 0.01 versus control group

To exclude the influence of different types of follicles, we also analyzed the average optical density of ApoC3 in different stages of follicles, and found that ApoC3 was obviously highly expressed in oocytes in primary follicles (PmF), secondary follicles (SF) and graafian follicles (GF) of PCOS mice (*P*<0.05, Fig. 6). However, no significant difference was observed in atretic follicles (AF) in the ovary of PCOS mice, compared to the controls. Based on these findings, we hypothesized that PCOS-related abnormal oocyte growth may be associated with overexpression of ApoC3.

### ApoC3 correlated with oocyte growth in postnatal healthy mice

**Fig. 7:**
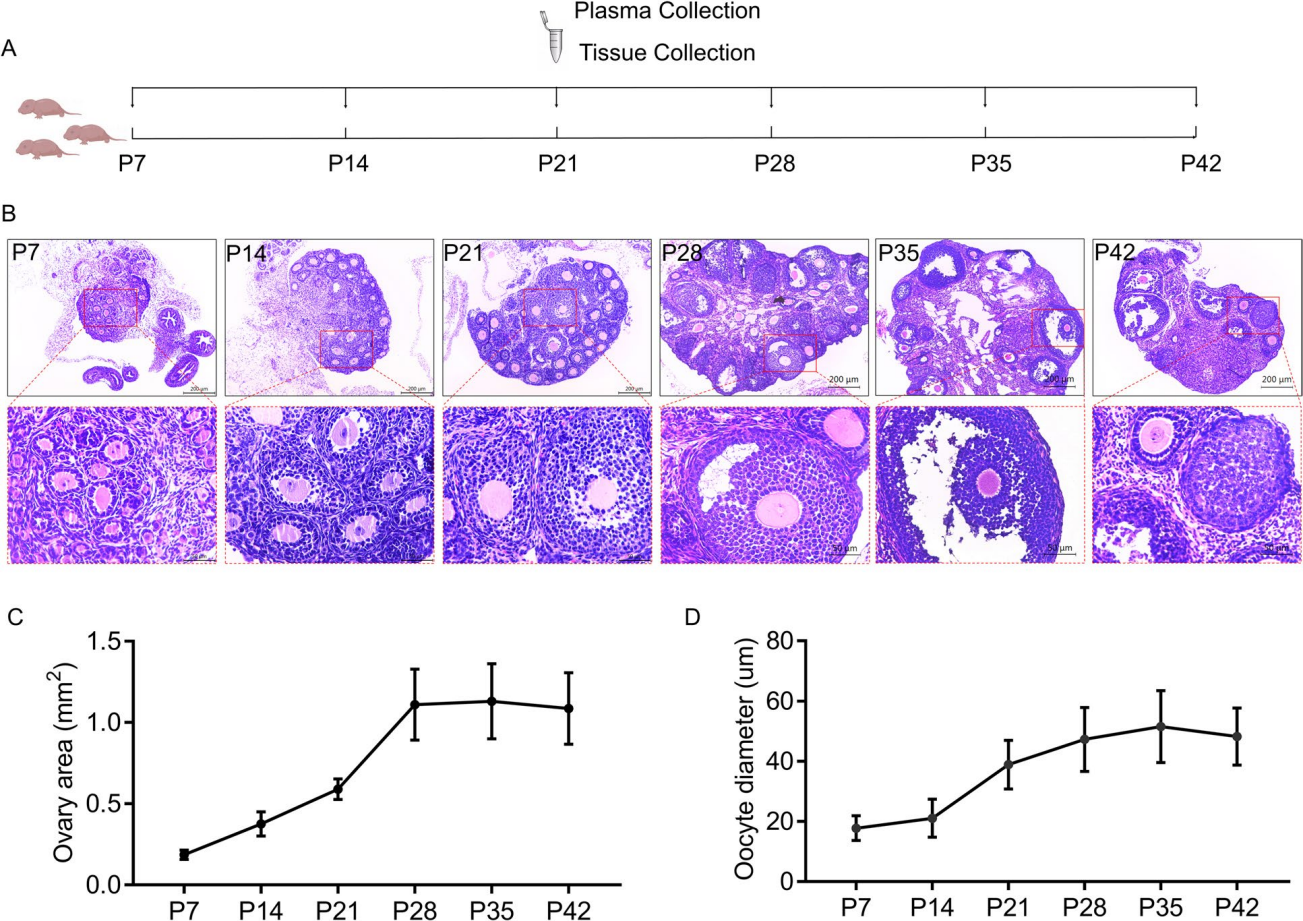
Histology of ovaries in healthy mice from P7 to P42 was described by HE staining. **A** Schematic representation of the experimental design whereby healthy mice in postnatal days of age 7 to 42 (P7-P42). **B** Representative HE staining of ovarian tissue from healthy mice from P7 to P42 (*n* = 6). Scale bar: 200 μm, 50 μm. **C** Area of ovarian tissue. **D** Oocyte diameter

**Fig. 8:**
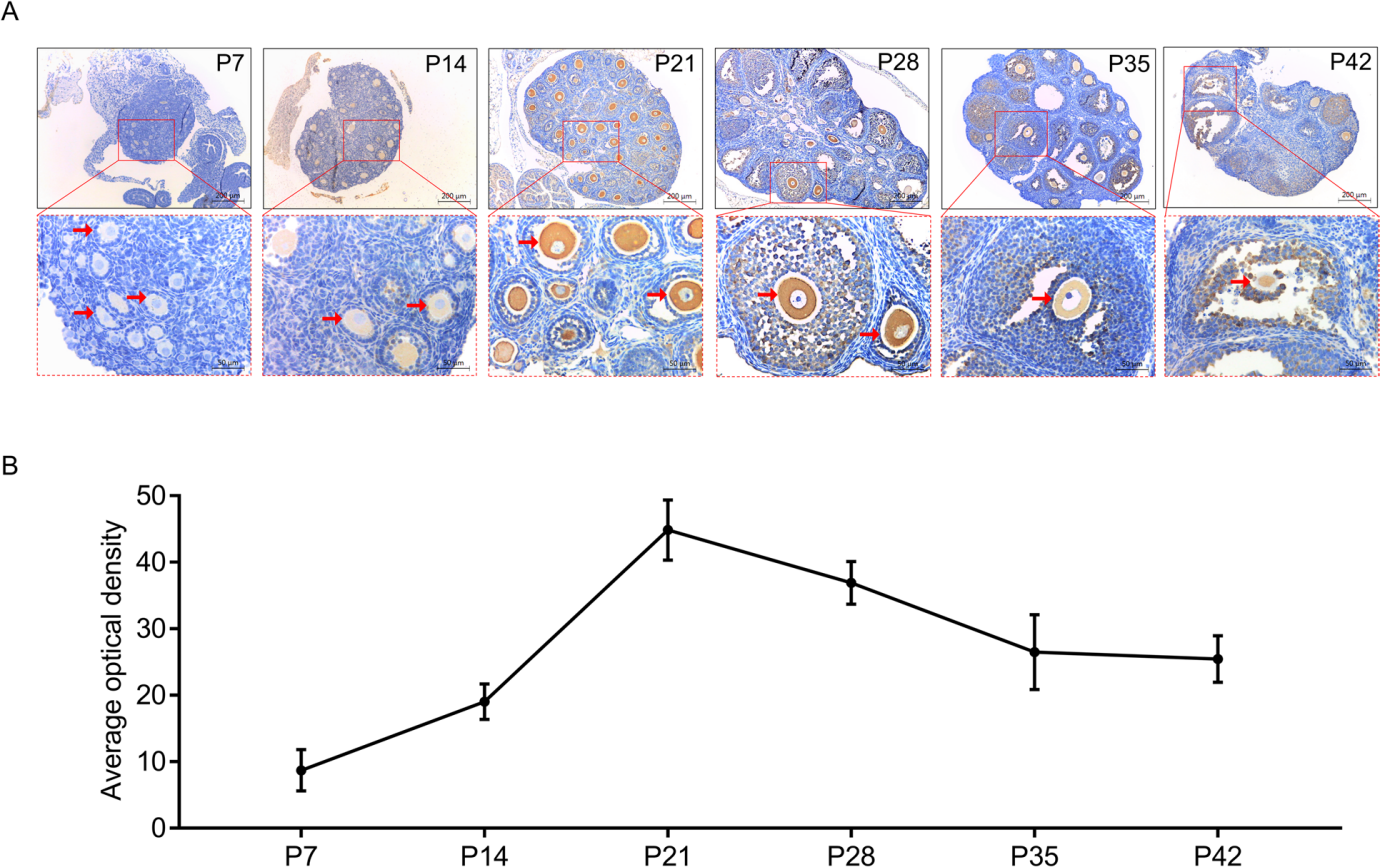
The presence of ApoC3 in oocyte ascended and arrived the peak on PD21 in healthy mice. **A** Immunohistochemical staining of ApoC3 in ovarian tissue from healthy mouse during P7 to P42 (*n* = 6). Area positive for ApoC3 was stained in brown; oocytes were indicated by red arrowheads. Scale bar: 200 μm, 50 μm. **B** Average optical density of ApoC3. Data were expressed as mean ± SD.

To further explore the expression of ApoC3 in healthy mice during postnatal development, we collected ovaries at postnatal age 7 (P7), P14, P21, P28, P35 and P42, as shown in Fig. 7A. Morphological analysis of the ovarian tissue was showed in Fig. 7B. The result demonstrated that ovary underwent a period of rapid development from P7-P28, after which development of ovary was stabilized from P28-P42 (Fig. 7C). The pattern of changes in oocyte diameter was similar to that of the ovary area with rapid development being observed from P14-P28 (Fig. 7D). We next investigated the changes of ApoC3 expression in healthy mouse ovaries from P7-P42. The examination revealed that expression of ApoC3 displayed a sharply increasing trend from P7 to P21, peaking at P21 and decreased then leveled off (Fig. 8).

## Discussion

Growing evidence suggests that apolipoprotein plays an essential role in the reproductive system of women [23], but the presence of ApoC3 in the ovary and its association with PCOS are largely unknown. In this study we reported for the first time that ApoC3 expresses in the ovarian tissue of PCOS patients and PCOS-like mouse. Importantly, the change in oocyte ApoC3 protein content was associated with PCOS development and plasma level of ApoC3 was related to sex hormone abnormalities in PCOS mice. Interestingly, we also found that the physiological elevation of ApoC3 in healthy mouse was closely associated with oocyte growth.

ApoC3 plays a critical role in the regulation of metabolism of free fatty acids by hindering the interaction between lipoprotein lipase and triglyceride-enriched lipoproteins, as well as their hydrolysis and clearance [24]. Fatty acids have the capacity to generate 3.5-fold more energy than glucose; thus, this form of metabolism for generating ATP is highly efficient [25]. ApoC3 influences lipid metabolism by moving from VLDL to HDL, with a transfer that is proportional to the magnitude of free fatty acid (FA) release [10]. Therefore, a certain level of ApoC3 is essential for oocyte metabolism. However, a rise in ApoC3 level could cause increased inhibition of lipoprotein lipase (LPL) activity and lead to lipolysis reduction and lipid deposition [10, 26]. Excessive lipid deposition would increase levels of reactive oxygen species (ROS), resulting in the dysfunction of mitochondria and endoplasmic reticulum, eventually impairing subsequent oocyte quality and development [27]. Therefore, a balanced level of ApoC3 in oocytes is necessary to ensure the developmental competence of oocytes [28]. In this regard, we found that the expression of ApoC3 was much stronger in the oocytes of POCS mice and PCOS women compared to the controls. This may be one of the reasons for the stagnation of PCOS follicle development and the deterioration of oocyte quality.

Thimoteus Speer, et al verified that ApoC3 activates the NLRP3 inflammasome in human monocytes by inducing an alternative NLRP3 inflammasome and impede endothelial regeneration and promote kidney injury [29]. What’s more ApoC3 overexpression aggravated early-stage diabetic nephropathy by activating the renal TLR2/NF-κB pathway with increased renal inflammation in mice [30]. Regarding the pathogenesis of PCOS, recent studies have intensively concentrated on the impact of chronic inflammation and low-grade inflammation playing a significant role in the emergence of ovulation problems in PCOS [31, 32]. Activation of the NLRP3 inflammasome accelerates ovarian fibrosis in mice with PCOS and plays an important role in regulating ovarian steroidogenesis, maturation of ovarian follicles, and other reproductive processes [33, 34]. Hence, we speculate that ApoC3 may be involved in the occurrence of ovarian inflammatory response in PCOS. However, the related studies between ApoC3 and inflammatory environment in PCOS are very scarce, further research in mechanism on ApoC3 and inflammation-related pathways in ovulation dysfunction of PCOS is an essential next step in confirming the findings.

To further explore the role of ApoC3 in oocyte during postnatal development, ovaries in P7-P42 mice were used in subsequent experiments. Our results revealed a remarkably fast rise in ApoC3 from P7 to P21, after which the expression declined. Evidence suggests that P22 to P23 is the “time window” for studying puberty initiation [35]. LH induces the synthesis of paracrine factors such as EGF-like factors and meiosis-activating sterol (MAS) to regulate oocyte germinal vesicle breakdown (GVBD) through cAMP/protein kinase A (PKA) and protein kinase C (PKC) pathways [36, 37]. After GVBD, meiosis resumes and homologous chromosomes are separated to emit the secondary oocyte and first polar body [38, 39]. Therefore, the interval of puberty initiation (P22 to P23) is a crucial phase in the control of mammalian oocyte meiosis. Here, we found that expression of ApoC3 in ovary of healthy mouse displayed a sharply increasing trend from P7 to P21, peaking at P21 and then decreased and flattened gradually. Thus, it seems that physiologically high expression of ApoC3 in the ovaries may possibly play a role in oocyte maturation, and likely acts as an indicative marker for oocyte development. However, unlike healthy mice, expression of ApoC3 elevated consistently in oocytes of PCOS-like mice. Thus, we speculate that ApoC3 may act as one of the vital factors that involve in the pathogenesis of PCOS.

Previous studies showed that elevated levels of several sex hormones, including LH, T and AMH, were associated with PCOS progression [40, 41]. Our study revealed an upward trend of ApoC3 in plasma that is positively correlated with LH, T and AMH during the progression of PCOS, but had no significant relationship to those hormones in the control group. Plasma lipoproteins are the major source of cholesterol for steroid production in the ovaries, the central core of the lipoprotein consists of cholesterol; apolipoprotein serves as a receptor recognition site that is closely related to cholesterol transportation and absorption [42, 43]. Cholesterol, as the precursor to steroid hormones in hormone production, is closely related to apolipoprotein [42–45]. However, only ApoE and ApoB-100 have been reported to act as key players in ovarian cholesterol uptake and steroidogenesis presently [23, 46]. AMH, as a relative of the transforming growth factor (TGF)-β family, plays an important biological role in follicle growth and oocyte development, directly correlates with PCOS, and profoundly impairs oocyte quality and maturation as well as embryo quality [47–49]. Higher AMH inhibits primordial follicle recruitment and aberrant follicular development [50, 51]. Moreover, the increased pulse frequency of LH stimulates theca cells to synthesize more androgens, resulting in the hyperandrogenic condition also inducing follicular arrest and ovulatory dysfunction [52]. Therefore, we speculated that increase level of ApoC3 in plasma has some unknown connection to ovulatory disturbances. However, rare relevant literatures related to ApoC3 and these hormones have been published. The relationship between ApoC3 and hormones is highly complex and poorly understood, which will be an intriguing and meaningful area for further study.

Finally, the role of apoC3 in insulin resistance deserves to be mentioned. Numerous studies suggest a close relationship exists between ApoC3 and insulin resistance [53]. An increased plasma level of ApoC3 in states associated with insulin resistance [54]. Under conditions of islet insulin resistance, local islet production of ApoC3 has been identified as a diabetogenic factor involved in increasing mitochondrial metabolism, deranging regulation of β-cell cytoplasmic free Ca2 + concentration ([Ca^2+^]i), and apoptosis for β-cell, which seems to link insulin resistance and β-cell failure in T2DM [55]. In addition, overexpression of ApoC3 enhances non-alcoholic fatty liver disease and exacerbates inflammatory pathways in skeletal muscles, affecting insulin signaling and thereby inducing insulin resistance [53]. Insulin resistance is a key pathophysiological marker of PCOS, insulin sensitizing agents have been used to improve insulin resistance in PCOS, and this may include beneficial effects on lipid and ApoB-lipoprotein metabolism [56–58]. In addition to peripheral insulin resistance, ovary insulin resistance will cause fertile disorder by having a side effect on folliculogenesis, causing anovulation and hyperandrogenia [59]. In our study, the correlation between ApoC3 levels and insulin in the plasma has not yet been found in PCOS mice. However, whether the high concentration of ApoC3 presents a key role in ovarian dysfunction and local insulin resistance are targets of future research in our next steps.

Taken together, we boldly speculated that distinct changes of ApoC3 expression in ovary of PCOS are associated with ovulation and oocyte quality. However, the data relevant to our validations is limited, whether ApoC3 can be produced within oocytes, and the causal relationship between high levels of ApoC3 and pathological changes of PCOS is the purpose of our following study. Although we have not been clear for the diagnosis and potential tailored management of infertility by ApoC3, it would be worthwhile to explore the application of ApoC3 as a biomarker in evaluation of infertility in women with PCOS [60, 61].

It should be noted that the clinical data presented in this study may have its limitation because of small sample sizes. Despite this, integrating clinical study and animal experiments is a strength of this study. In addition, the PCOS mouse model was induced by injecting DHEA in our research and thus it should be confirmed this factor may have an impact on subsequent results. Further investigations are required to study the causes of abnormally high expression of ApoC3 in this PCOS mouse model, and deeply explore the mechanisms of ApoC3 in PCOS.

## Conclusions

Our study showed that the increased ApoC3 in ovary of PCOS located in oocyte may involve the progression of ovulation disorder. We also found a peak of ApoC3 in mouse oocytes in postnatal development age, indicating that ApoC3 may also be involved in oocyte development. These findings suggest that elevated ovarian ApoC3 level might play a key role in PCOS development and normal oocyte growth.

## Materials and methods

### Human subjects

In this study, ovary tissue and follicular fluid samples are used for analysis. A total of 12 PCOS patients and 12 controls underwent surgery in Guangdong Women and Children Hospital from January 2014 to January 2015 were included in this study for ovarian tissue sampling. Fourteen PCOS patients and thirteen control women who underwent conventional in vitro fertilization (IVF) between December 2020 and March 2021 were included for follicular fluid analysis.

For the PCOS group, the inclusion criteria were: (i) age 18 to 35 years; (ii) a confirmed diagnosis of PCOS based on the Rotterdam diagnostic criteria of 2003 [62]; (iii) participants underwent IVF/ICSI or surgical wedge resection under laparoscopy. For the control group, the inclusion criteria were: (i) age 18–35 and BMI- matched subjects; (ii) patients with regular menstrual cycles, normal ovary sonographs and normal ovulating; (iii) participants underwent IVF/ICSI or resected to treat a unilateral benign ovarian teratoma or an ovarian cyst. The exclusion criteria were as follows: participants had a history of diabetes or other endocrinological disease, such as premature ovarian failure, hyperprolactinemia, adrenal hyperplasia, cushing’s syndrome, thyroid disorders, severe mental illness, or ovarian tumors.

Ovarian tissue and follicular fluid sampling was performed as previously described [63–65]. Venous blood samples were collected during the early follicular phase of menstrual bleeding (morning on the 3rd − 5th days). All subjects gave their informed consent for inclusion before they participated in the study. The study was conducted in accordance with the Declaration of Helsinki, and the protocol was approved by the Ethics and Research Committee of Guangdong Women and Children Hospital (No. 201401011; 201901012).

### Animals experiment

All mice were housed in the animal facility of SPF Animal Laboratory (License number: SYXK 2019 − 0144) with a temperature of 22 ± 1°C and 12-h light–dark cycle. Mice were allowed to eat and drink freely. Animal experiments were approved by Guangzhou University of Chinese Medicine Animal Care and Use Committee (No. 20220504; No. 20220719), and conducted according to fundamental principles of Basel Declaration and the International Council for Laboratory Animal Science (ICLAS).

Three-week-old C57BL/6 mice were randomly divided into 2 groups (*n* = 18 for each group) and were injected with dehydroisoandrosterone (DHEA, Macklin, Shanghai, China)(6 mg/100 g body weight) and sesame oil (Macklin, Shanghai, China) for 21 consecutive days respectively as described previously [66]. All mice were anesthetized with 3% isoflurane (ISO) (in 40% oxygen) and were euthanized by bloodletting prior to dissection at preset time intervals. Mice in postnatal days of age 7 to 42 (P7-P42) were divided into six groups according to age.

### Estrous cycle determination

Vaginal smears of all mice were taken daily from the 10 days after the first injection for 10 consecutive days to observe the oestrus cycle. PBS-wetted cotton swabs were used to collect vaginal cells, and the liquid was applied to slides to dry. The sections were stained with Wright-Giemsa dye (Leagene Biotechnology, Beijing, China) [67].

### Detection of animals plasma biochemical indicators

The levels of ApoC3 (Cloud-Clone Corp, Texas, USA, SEB890Mu), testosterone (Demeditec Diagnostics, Kiel, German, DE1559), luteinizing hormone (Nanjing Jian Cheng Bioengineering Institute, Nanjing, China, H206-1-2), follicle stimulating hormone (Cloud-Clone Corp, Texas, USA, CEA830Mu), anti-müllerian hormone (Cloud-Clone Corp, Texas, USA, CEA228Mu) and insulin (Mercodia, Uppsala, Sweden, 10-1247-01) were determined using enzyme-linked immunosorbent assay kits on a spectrophotometer (ThermoFisher, Singapore). The levels of triglyceride, total cholesterol, low-density lipoprotein cholesterol and high-density lipoprotein cholesterol (Nanjing Jian Cheng Bioengineering Institute, Nanjing, China) were examined using biochemical analysis kits in accordance with the instructions provided by the manufacturers.

### Glucose tolerance test

Mice were fasted for 12 h before the glucose tolerance test (GTT) experiment. Glucose levels were measured by an Accu-Chek Performa blood glucose meter (Roche Diagnostics, Mannheim, Germany) on a tail vein blood sample. After measurement of fasting glucose levels. The mice were orally gavaged with D-glucose (2g/kg body weight) for GTT and tail sampling was performed at time points 15, 30, 60, 90 and 120 min after the administration for glucose detection [68].

### Histomorphological analysis

The paraffin-embedded tissue was sequentially dehydrated with gradient ethanol and penetrated by xylene and embedded in paraffin. The sections were longitudinally sliced into 3 µm slices (Leica, Nussloch, Germany), dewaxed in xylene and rehydrated with gradient ethanol, then were stained with hematoxylin and eosin dye, and mounted using neutral resin. The numbers of corpora lutea and cystic follicles were counted under a microscope (Leica, Wetzlar, Germany). The area of all sections derived from the same ovary was determined, and the maximum value was selected as the area of the ovary; oocyte diameter was calculated using the mean of two perpendicular measurements.

### Western blot

Total protein of mouse ovaries for each sample was extracted using RIPA buffer (CST, Beverly, Massachusetts, USA) containing protease inhibitor cocktail (Roche, Basel, Switzerland). The protein samples were electrophoretically separated using 12–15% SDS-PAGE and transferred to PVDF membranes (Bio-Rad, Hercules, CA, USA). The membranes were placed in 5% BSA for 1 h to block nonspecific binding and then incubated with the corresponding primary antibodies (ApoC3: Servicebio, Wuhan, China; GAPDH: Santa, USA) for 12 h at 4°C, and then incubated for 1 h with the corresponding secondary antibodies at room temperature. Finally, the membranes were visualized using chemiluminescence (ECL) reaction reagents (GBCBIO Technologies Inc. Guangzhou, China) and imaged by the ECL system (Cell Biosciences, USA). The band density was quantified using ImageJ software (NIH Image, Bethesda, MD, USA).

### RNA extraction and qRT-PCR

Total RNA was isolated according TRIzol reagent (Invitrogen, Carlsbad, CA), and cDNA was made RevertAid First Strand cDNA Synthesis Kit (ThermoFisher, USA). Quantitative real-time PCR was carried out by monitoring the increase in fluorescence of SYBR green with the Real Time PCR System (Applied Biosystems, CA, USA). The primer sequences (5’-3’) were obtained from PrimerBank (https://pga.mgh.harvard.edu/primerbank/) and synthesized in Invitrogen: β-actin (F) TGAGCTGCGTTTTACACCCT, (R) GCCTTCACCGTTCCAGTTTT; ApoC3 (F) GCTCAGTTTTATCCCTAGAAGCA, (R) AGTGATTGTCCATCCAGCCC. The 2^−ΔΔCt^ method was used to calculate relative changes in mRNA expression and normalized with β-actin.

### Immunohistochemistry staining

The ovarian tissues were embedded in paraffin, cut into 3-µm sections and dewaxed as described above. Antigen retrieval was performed in pH 6.0 sodium citrate buffer in a microwave at 100° C and the endogenous peroxidases were quenched with 3% H_2_O_2_. The sections were blocked with normal goat serum for 10 min at room temperature and then incubated with primary antibodies ApoC3 (Bioss, Beijing, China, for human; Servicebio, Wuhan, China, for mouse). The biotin-labeled sheep anti-rabbit IgG antibody was added to sections and incubated at room temperature. After washing with PBS, streptavidin-HRP was added and the chromogenic reaction was achieved with DAB mixed liquid (CWBio, Jiangsu, China). The semi-quantitative analysis of ApoC3 was conducted by calculating the average optical density (AOD) using IHC Profiler by Image J software (the National Institutes of Health, Bethesda, MD, USA) [69, 70].

### Immunofluorescence analysis

The sections were blocked with normal goat serum for 10 min at room temperature, incubated with ApoC3 antibodies (Servicebio, Wuhan, China) for 12 h at 4°C, and then incubated with Alexa Fluor 594-conjugated goat anti-rabbit IgG secondary antibody (Jackson ImmunoResearch, West Grove, USA) for 1 h at room temperature, protected from light. Finally, the slides were mounted on an antifade mounting medium containing DAPI as a counter-stain (Beyotime, Shanghai, China) at room temperature for 1 min. Sections were photographed using an epifluorescence inverted microscope (Carl-Zeiss, Jena, Germany), and the relative fluorescence intensity was calculated by dividing the fluorescence intensity of cells by the fluorescence intensity of the background using Image J software.

### Statistical analysis

Statistical calculations were performed using SPSS 25 (IBM Corp., Armonk, NY) and the data were expressed as mean ± standard deviation. Graphs were generated by using GraphPad Prism 7 (Dotmatics, Inc). All the data conformed to a normal distribution, as determined by Q-Q plots. Comparisons between two groups were evaluated using independent-sample t tests preformed by SPSS 25. Prior to the t-test, an F-test was used to determine equal or unequal variance. If the F-value was more than 0.05, then P-values were calculated using a Student’s t-test of equal variance. If the F-value was less than 0.05, then a Student’s t-test of unequal variance was used.. Pearson correlation coefficient was used to determine the relation between the variables of sex hormones and ApoC3. A results of *P < 0.05* was considered statistically significant.

### Supplementary Information

**Additional file 1: Supplementary Materials Table S1.** Pearson’s correlations ApoC3 levels and hormone parameters in PCOS mouse models and control group (**Table S2**)

## Data Availability

All data generated or analysed during this study are included in this published article [and its supplementary information files].

## References

[1] Siddiqui S, Mateen S, Ahmad R, Moin S (2022). A brief insight into the etiology, genetics, and immunology of polycystic ovarian syndrome (PCOS). J Assist Reprod Genet.

[2] Liu Q, Jiang J, Shi Y, Mo Z, Li M (2020). Apelin/Apelin receptor: a new therapeutic target in polycystic ovary syndrome. Life Sci.

[3] Chakraborty P, Goswami SK, Rajani S, Sharma S, Kabir SN, Chakravarty B (2013). Recurrent pregnancy loss in polycystic ovary syndrome: role of hyperhomocysteinemia and insulin resistance. PLoS ONE.

[4] Zhang X, Miao H, Zhou J, Chen Y, Ou Y, Song Y (2023). Association between preconception anti-androgen therapy and pregnancy outcomes of patients with PCOS: a prospective cohort study. Front Endocrinol (Lausanne).

[5] Li Y, Chen C, Ma Y, Xiao J, Luo G, Li Y (2019). Multi-system reproductive metabolic disorder: significance for the pathogenesis and therapy of polycystic ovary syndrome (PCOS). Life Sci.

[6] Kakoly NS, Khomami MB, Joham AE, Cooray SD, Misso ML, Norman RJ (2018). Ethnicity, obesity and the prevalence of impaired glucose tolerance and type 2 diabetes in PCOS: a systematic review and meta-regression. Hum Reprod Update.

[7] Teede HJ, Misso ML, Costello MF, Dokras A, Laven J, Moran L (2018). Recommendations from the international evidence-based guideline for the assessment and management of polycystic ovary syndrome. Fertil Steril.

[8] Escobar-Morreale HF (2018). Polycystic ovary syndrome: definition, aetiology, diagnosis and treatment. Nat Rev Endocrinol.

[9] Li L, Zhang J, Zeng J, Liao B, Peng X, Li T (2020). Proteomics analysis of potential serum biomarkers for insulin resistance in patients with polycystic ovary syndrome. Int J Mol Med.

[10] Norata GD, Tsimikas S, Pirillo A, Catapano AL (2015). Apolipoprotein C-III: from pathophysiology to Pharmacology. Trends Pharmacol Sci.

[11] Sztolsztener K, Zywno H, Hodun K, Konończuk K, Muszyńska-Rosłan K, Latoch E (2022). Apolipoproteins-new biomarkers of overweight and obesity among Childhood Acute Lymphoblastic Leukemia Survivors?. Int J Mol Sci.

[12] Schunk SJ, Hermann J, Sarakpi T, Triem S, Lellig M, Hahm E (2021). Guanidinylated Apolipoprotein C3 (ApoC3) associates with kidney and vascular Injury. J Am Soc Nephrol.

[13] Kanter JE, Shao B, Kramer F, Barnhart S, Shimizu-Albergine M, Vaisar T (2019). Increased apolipoprotein C3 drives cardiovascular risk in type 1 diabetes. J Clin Invest.

[14] D’Erasmo L, Di Costanzo A, Gallo A, Bruckert E, Arca M (2020). ApoCIII: a multifaceted protein in cardiometabolic disease. Metabolism.

[15] Kumariya S, Ubba V, Jha RK, Gayen RJ (2021). Autophagy in ovary and polycystic ovary syndrome: role, dispute and future perspective. Autophagy.

[16] Emanuel R, Roberts J, Docherty PD, Lunt H, Campbell RE, Möller K (2022). A review of the hormones involved in the endocrine dysfunctions of polycystic ovary syndrome and their interactions. Front Endocrinol (Lausanne).

[17] Mansour A, Hashemi Taheri AP, Moradi B, Mohajeri-Tehrani MR, Qorbani MQ, Pashakolaee SG (2022). Ovarian volume, not follicle count, is independently associated with androgens in patients with polycystic ovary syndrome. BMC Endocr Disord.

[18] Jozkowiak M, Piotrowska-Kempisty H, Kobylarek D, Gorska N, Mozdziak P, Kempisty B (2022). Endocrine disrupting chemicals in polycystic ovary syndrome: the relevant Role of the Theca and Granulosa cells in the pathogenesis of the ovarian dysfunction. Cells (Basel Switzerland).

[19] Kim YJ, Cho YI, Jang J, Koo YD, Park SW, Lee JH (2023). Lovastatin, an Up-Regulator of low-density lipoprotein receptor, enhances Follicular Development in Mouse Ovaries. Int J Mol Sci.

[20] Shamsi M, Ganji A, Mosayebi G, Amirhoseiny ES, Shohani S, Ghazavi A (2023). Chamomile and Urtica dioica extracts improve immunological and histological alterations associated with polycystic ovarian syndrome in DHEA -induced mice. BMC Complement Med Ther.

[21] Corrie L, Gulati M, Singh SK, Kapoor B, Khursheed R, Awasthi A (2021). Recent updates on animal models for understanding the etiopathogenesis of polycystic ovarian syndrome. Life Sci.

[22] Mimouni NEH, Paiva I, Barbotin A, Timzoura FE, Plassard D, Gras SL (2021). Polycystic ovary syndrome is transmitted via a transgenerational epigenetic process. Cell Metab.

[23] Oria RB, de Almeida JZ, Moreira CN, Guerrant RL, Figueiredo JR (2020). Apolipoprotein E Effects on mammalian ovarian steroidogenesis and human fertility. Trends Endocrinol Metab.

[24] Sathiyakumar V, Kapoor K, Jones SR, Banach M, Martin SS, Toth PP (2018). Novel therapeutic targets for managing Dyslipidemia. Trends Pharmacol Sci.

[25] Richani D, Dunning KR, Thompson JG, Gilchrist RB (2021). Metabolic co-dependence of the oocyte and cumulus cells: essential role in determining oocyte developmental competence. Hum Reprod Update.

[26] Giammanco A, Spina R, Cefalu AB, Averna M (2023). APOC-III: a gatekeeper in Controlling triglyceride metabolism. Curr Atheroscler Rep.

[27] Prates EG, Nunes JT, Pereira RM (2014). A role of lipid metabolism during cumulus-oocyte complex maturation: impact of lipid modulators to improve embryo production. Mediators Inflamm.

[28] Khan R, Jiang X, Hameed U, Shi Q (2021). Role of lipid metabolism and signaling in mammalian oocyte maturation, Quality, and Acquisition of competence. Front Cell Dev Biol.

[29] Zewinger S, Reiser J, Jankowski V, Alansary D, Hahm E, Triem S (2020). Apolipoprotein C3 induces inflammation and organ damage by alternative inflammasome activation. Nat Immunol.

[30] Wang H, Huang X, Xu P, Liu X, Zhou Z, Wang F (2021). Apolipoprotein C3 aggravates diabetic nephropathy in type 1 diabetes by activating the renal TLR2/NF-kappaB pathway. Metabolism.

[31] Wang J, Yin T, Liu S (2023). Dysregulation of immune response in PCOS organ system. Front Immunol.

[32] Rudnicka E, Suchta K, Grymowicz M, Calik-Ksepka A, Smolarczyk K, Duszewska AM (2021). Chronic low Grade inflammation in Pathogenesis of PCOS. Int J Mol Sci.

[33] Zhou F, Li C, Zhang S (2020). NLRP3 inflammasome: a new therapeutic target for high-risk reproductive disorders?. Chin Med J (Engl).

[34] Wang D, Weng Y, Zhang Y, Wang R, Wang T, Zhou J (2020). Exposure to hyperandrogen drives ovarian dysfunction and fibrosis by activating the NLRP3 inflammasome in mice. Sci Total Environ.

[35] Chen Y, Liu Q, Liu R, Yang C, Wang X, Ran Z (2021). A Prepubertal mice Model to study the growth pattern of early ovarian follicles. Int J Mol Sci.

[36] Hua R, Liu M (2021). Sexual dimorphism in mouse meiosis. Front Cell Dev Biol.

[37] Pan B, Li J (2019). The art of oocyte meiotic arrest regulation. Reprod Biol Endocrinol.

[38] Zhang M, Xia G (2012). Hormonal control of mammalian oocyte meiosis at diplotene stage. Cell Mol Life Sci.

[39] Sun QY, Miao YL, Schatten H (2009). Towards a new understanding on the regulation of mammalian oocyte meiosis resumption. Cell Cycle.

[40] Zhang H, Wang W, Zhao J, Jiao P, Zeng L, Zhang H (2023). Relationship between body composition, insulin resistance, and hormonal profiles in women with polycystic ovary syndrome. Front Endocrinol (Lausanne).

[41] Teede H, Misso M, Tassone EC, Dewailly D, Ng EH, Azziz R et al. Anti-Müllerian hormone in PCOS: a Review Informing International Guidelines. Trends Endocrinol Metab. 2019:30(7):467–78. DOI: 10.1016/j.tem.2019.04.006. 10.1016/j.tem.2019.04.006 31160167

[42] Hieronimus B, Griffen SC, Keim NL, Bremer AA, Berglund L, Nakajima K (2019). Effects of Fructose or glucose on circulating ApoCIII and triglyceride and cholesterol content of Lipoprotein Subfractions in humans. J Clin Med.

[43] Wang M, Zhao D, Xu L, Guo W, Nie L, Lei Y (2019). Role of PCSK9 in lipid metabolic disorders and ovarian dysfunction in polycystic ovary syndrome. Metabolism.

[44] Liang J, Zhang B, Hu Y, Na Z, Li D (2023). Effects of steroid hormones on lipid metabolism in sexual dimorphism: a mendelian randomization study. Front Endocrinol (Lausanne).

[45] Martinez MN, Emfinger CH, Overton M, Hill S, RamaswamyL TS, Cappel DA (2012). Obesity and altered glucose metabolism impact HDL composition in CETP transgenic mice: a role for ovarian hormones. J Lipid Res.

[46] Dyer CA, Curtiss LK (1988). Apoprotein E-rich high density lipoproteins inhibit ovarian androgen synthesis. J Biol Chem.

[47] Howard JA, Hart KN, Thompson TB (2022). Molecular Mechanisms of AMH Signaling. Front Endocrinol (Lausanne).

[48] Muharam R, Prasetyo YD, Prabowo KA, Putri YI, Maidarti M, Hestiantoro A (2022). IVF outcome with a high level of AMH: a focus on PCOS versus non-PCOS. BMC Womens Health.

[49] Liu X, Han Y, Wang X, Zhang Y, Du A, Yao R (2022). Serum anti-mullerian hormone levels are associated with early miscarriage in the IVF/ICSI fresh cycle. BMC Pregnancy Childbirth.

[50] Cedars MI (2022). Evaluation of female Fertility-AMH and Ovarian Reserve Testing. J Clin Endocrinol Metab.

[51] Peluso C, Fonseca FLA, Rodart IF, Cavalcanti V, Gastaldo G, Christofolini DM (2014). AMH: an ovarian reserve biomarker in assisted reproduction. Clin Chim Acta.

[52] Liao B, Qiao J, Pang Y (2021). Central Regulation of PCOS: abnormal Neuronal-Reproductive metabolic circuits in PCOS Pathophysiology. Front Endocrinol (Lausanne).

[53] Christopoulou E, Tsimihodimos V, Filippatos T, Elisaf M (2019). Apolipoprotein CIII and diabetes. Is there a link?. Diabetes Metab Res Rev.

[54] Borén J, Packard CJ, Taskinen M (2020). The roles of ApoC-III on the metabolism of triglyceride-rich lipoproteins in humans. Front Endocrinol (Lausanne).

[55] Avall K, Ali Y, Leibiger IB, Leibiger B, Moede T, Paschen M (2015). Apolipoprotein CIII links islet insulin resistance to beta-cell failure in diabetes. Proc Natl Acad Sci U S A.

[56] Kupreeva M, Diane A, Lehner R, Watts R, Ghosh M, Proctor S (2019). Effect of metformin and flutamide on insulin, lipogenic and androgen-estrogen signaling, and cardiometabolic risk in a PCOS-prone metabolic syndrome rodent model. Am J Physiol Endocrinol Metab.

[57] Paul C, Lagana AS, Maniglio P, Triolo O, Brady DM (2016). Inositol’s and other nutraceuticals’ synergistic actions counteract insulin resistance in polycystic ovarian syndrome and metabolic syndrome: state-of-the-art and future perspectives. Gynecol Endocrinol.

[58] Laganà AS, Rossetti P, Buscema M, Vignera SL, Condorelli RA, Gullo G (2016). Metabolism and ovarian function in PCOS Women: a Therapeutic Approach with Inositols. Int J Endocrinol.

[59] Heber MF, Ferreira SR, Abruzzese GA, Raices T, Pignataro OP, Vega M et al. Metformin improves ovarian insulin signaling alterations caused by fetal programming. J Endocrinol. 2019, JOE-18-0520.R1. DOI: 10.1530/JOE-18-0520. 10.1530/JOE-18-0520 30620715

[60] Lagana AS, Uccella S, Chiantera V, Garzon S (2022). Molecular Biology of Human Fertility: stepping towards a tailored Approach. Int J Mol Sci.

[61] Mikus M, Goldstajn MS, Brlecic I, Dumancic S, Lagana AS, Chiantera V (2022). CTLA4-Linked autoimmunity in the pathogenesis of endometriosis and related infertility: a systematic review. Int J Mol Sci.

[62] Joham AE, Norman RJ, Stener-Victorin E, Legro RS, Franks S, Moran LJ (2022). Polycystic ovary syndrome. Lancet Diabetes Endocrinol.

[63] Li L, Zhang J, Deng Q, Li J, Li Z, Xiao Y (2016). Proteomic profiling for identification of novel biomarkers differentially expressed in human ovaries from polycystic ovary syndrome patients. PLoS ONE.

[64] Li L, Mo H, Zhang J, Zhou Y, Peng X, Luo X (2016). The role of heat shock protein 90B1 in patients with polycystic ovary syndrome. PLoS ONE.

[65] Ding Y, Jiang Y, Zhu M, Zhu Q, He Y, Lu Y (2022). Follicular fluid lipidomic profiling reveals potential biomarkers of polycystic ovary syndrome: a pilot study. Front Endocrinol (Lausanne).

[66] Ma R, Wang S, Xue M, Zhang H, He Z, Jueraitetibaike K (2023). Effects of n-3 PUFA supplementation on oocyte in vitro maturation in mice with polycystic ovary syndrome. J Ovarian Res.

[67] McLean AC, Valenzuela N, Fai S, Bennett SAL (2012). Performing vaginal lavage, crystal violet staining, and vaginal cytological evaluation for mouse estrous cycle staging identification. J Vis Exp.

[68] Virtue S, Vidal-Puig A (2021). GTTs and ITTs in mice: simple tests, complex answers. Nat Metab.

[69] Meng M, Tan J, Chen W, Du Q, Xie B, Wang N (2019). The fibrosis and immunological features of Hypochlorous Acid Induced Mouse Model of systemic sclerosis. Front Immunol.

[70] Varghese F, Bukhari AB, Malhotra R, De A (2014). IHC profiler: an open source plugin for the quantitative evaluation and automated scoring of immunohistochemistry images of human tissue samples. PLoS ONE.

