# Supplementary material for: ApoC3 is expressed in oocytes and increased expression is associated with PCOS progression
Source: J Ovarian Res. 2023 Sep 9;16:188. doi: 10.1186/s13048-023-01263-6 (PMC10493025; doi:10.1186/s13048-023-01263-6)
Supplement: Supplementary file 1 — Additional file 1: Supplementary Materials Table S1. Pearson’s correlations ApoC3 levels and hormone parameters in PCOS mouse models and control group (Table S2) [file 13048_2023_1263_MOESM1_ESM.docx]

**Table 1. Correlation coefficients between ApoC3 levels and hormone parameters in mouse models for PCOS.**

| Pearson Correlation Coefficient (R) | LH  (mUmL) | FSH  (ng/mL) | T  (ng/mL) | AMH  (pg/mL) | ApoC3  (μg/mL) |
| --- | --- | --- | --- | --- | --- |
| LH (mUmL) | 1 |  |  |  |  |
| FSH (ng/mL) | -0.272 | 1 |  |  |  |
| T (ng/mL) | 0.747** | 0.179 | 1 |  |  |
| AMH (pg/mL) | 0.782** | -0.152 | 0.749** | 1 |  |
| ApoC3 (μg/mL) | 0.709** | -0.442 | 0.627** | 0.680** | 1 |

LH: lutein stimulating hormone; FSH: follicle stimulating hormone; T: testosterone; AMH: anti-mullerian hormone; ApoC3: apolipoprotein C3. Correlation analysis was performed with Pearson correlation test. **P*<0.05, ***P*<0.01 and ****P*<0.001.

**Table 2. Correlation coefficients between ApoC3 levels and hormone parameters in control mice.**

| Pearson Correlation  Coefficient (R) | LH  (mUmL) | FSH  (ng/mL) | T  (ng/mL) | AMH  (pg/mL) | ApoC3  (μg/mL) |
| --- | --- | --- | --- | --- | --- |
| LH (mUmL) | 1 |  |  |  |  |
| FSH (ng/mL) | 0.671* | 1 |  |  |  |
| T (ng/mL) | 0.547 | 0.208 | 1 |  |  |
| AMH (pg/mL) | -0.275 | 0.496 | 0.044 | 1 |  |
| ApoC3 (μg/mL) | -0.172 | -0.227 | -0.263 | 0.028 | 1 |

LH: lutein stimulating hormone; FSH: follicle stimulating hormone; T: testosterone; AMH: anti-mullerian hormone; ApoC3: apolipoprotein C3. Correlation analysis was performed with Pearson correlation test. **P*<0.05.
